# Circumcision Enforced by Law

**Published:** 1889-07

**Authors:** J. M. Vandavel

**Affiliations:** Waco, Texas


For Daniel's Texas Medical Journal.

### CIRCUMCISION ENFORCED BY LAW.

BY J. M. VANDAVEL, M. D., WACO.

[Read at Convention Lone State Medical Society, Houston, June 25, 1889.]

WHILE we agree in a measure with the statement that the world is governed too much by prohibitory or implied prohibitory acts, we must also agree in the fact that governments are organized, fostered and perpetuated for the general welfare of the constituency, individually and collectively.

It is the duty of the civic or municipal, state and national governments to look after the *habits* and *customs* of their citizens respectively, and direct in a legal way *whenever* it is apprehended, or discovered, that general prosperity is crippled, general health injured and the sanitary status are of such a character as to impede sanitary invironment (and healthfulness); dwarf the civilian in physique and thought; degenerate robust muscularity and mental acumen, legislative action becomes as paramount as the quarantine to prevent yellow fever, small-pox, cholera asiatica, and as absolutely necessary as the interstate commerce bill to prevent discriminations in freight rates and railroad travel; because these affect the general welfare.

It is a fact known to all and doubted by few, if any at all, that the rapid advancement of the most learned science during the past three decades has been almost phenomenal, and to speak of the various indices showing this progress is too laborious for the hour, too comprehensive for your servant to grapple with on this occasion.

Going back three thousand years, many years before the fathers of medicine were born, to the days of the Astrologer, we present you with a rite as old as the Bible: the taking of the "fore-skin" of each male child; and in Egypt the circumcision of females was also practiced.

We trust that each scientific mind will lay aside his orthodox teaching on our subject and view it primarily from a medical, or more specifically, as a sanitary measure or necessity. In all past ages of the world's history, as well as the present, the Hebrew has stood as impregnable to the common scourges and devastation which have swept away the earth's inhabitants that we have been forced to ejaculate, "God's peculiar people truly."

A lower mortality than any race now on the face of the earth (in proportion to number), and this of course means longer life on the average; freedom or comparative freedom from the diseases which prey upon the common herd of humanity, viz., tubercle, phthisis pulmonalis, adenitis, carcinoma, scrofula, rachitis and venereal cachexia and of all diseases constitutional in their terrible ravages which come in direct harmony with and in obeisance to that scriptural injunction which says "the sins and iniquities of the fathers shall visit the children unto the third and fourth generation;" and until the malignancy of these blood taints shall have been exhausted in destroying general health and happiness, and depopulating some of the most densely inhabited sections of the southern states.

It is a fact found in my own every day work and in conversation, and by reading the scanty literature on this subject that out of every five males suffering from venereal taint, four will have the long prepuce.

Besides, not adding anything to the looks of the organ, this elongated prepuce in the first place renders venereal disease much easier caught; and in the second place makes it decidedly much worse to treat.

This proposition being granted as true, the question of the enforcement of circumcision is the one most needed to be discussed. That circumcision should be enforced by law only obtains in so far as we can show that consumption, scrofula, glandular enlargements, rheumatism, heart trouble and eczema are sequalæ in any manner of syphilis.

The transmission of syphilis is one of the medical axioms; i. e., syphilis often pure and simple in the offspring—often syphilis in a modified form, for we see daily, syphilis transmitted and assuming the form of dermal eruptions, osseous dyscrasia, deformations, and in whatever way this taint manifests itself, it carries along with it deteriorations of a physique which otherwise might have been an admirable specimen of God's handiwork. If we can check the ravages of disease, cut off inherited diseases transmitted by marriage and illegitimate cohabitation, improve general health and happiness, calm excited brain and vaso-moto nerve centers, sooth supra reflex nerve excitation, we will have, in a measure, cured in advance many diseases and imagined diseases, both of mind and body, originating in an elongated prepuce, bringing on onanism, early cohabitation, phimosis, paraphimosis, and syphilis and sequalæ. Simultaneous with syphilitic poison in the organ, every gland in the body is often more or less excited by metastisis, and the glands often breaking down or suppurating, and especially the inguinal and the cervical.

The good such a law would bring to the people of this country, especially the colored people of the South land, where virtue is so slovenly and loosely worn by our women, morality so little valued, and the marriage relationship so easily adulterated, or sullied, can be better appreciated by comparing the American races with the Sandwich Islanders. It is said that one hundred years ago there were (400,000) four hundred thousand inhabitants on these islands, healthy, prolific, and, in their peculiar way, prosperous; but since the importation and propagation of syphilis amongst that people, they have dwindled to (40,000) forty thousand, with a death rate equal to, if not exceeding, the birth rate; and as a matter of fact, it will be only a few decades when these people must become extinct, unless a check is made to the depopulating disease which is now threatening them, or unless the islands are peopled by a foreign element whose mode of living will be within the limits of a sanitary surveilliance worthy of the name.

Beside a very heavy death rate, there is another factor working against the islanders, and that is, sterility, which is common to a very large number, both male and female, owing primarily to the thorough work of this disease, which has been described as "riding a man through the world and then jumping on his ghost and riding it through eternity."

It is but right, looking these facts sternly in the face, for the government to look after the welfare of the races, black and white, and especially our own race, because as yet the race is infantile in the correct mode of living, ignorant of sanitary laws, and often heedless when known, and blind yet as to the daily and inexorable laws of nature to which all humanity must conform in order to be healthy and happy; the importance of this the race has not yet been taught.

The Jew is well provided for. His dietary is on a higher and more wholesome basis than the most fastidious American can boast of, and he sees that his food is not only prepared well for the table, but that it is killed scientifically. They hold circumcision in high esteem only as an ordinance enjoined as a sanitary necessity—a health safeguard.

At the present rapidity by which the venereal taint is being propagated among the colored people, even mere children being the worst sufferers, owing to fear and an attempt at secrecy, it will only be a matter of time when we will wish to call a halt; but it will be to late.

